# The PPE Domain of PPE17 Is Responsible for Its Surface Localization and Can Be Used to Express Heterologous Proteins on the Mycobacterial Surface

**DOI:** 10.1371/journal.pone.0057517

**Published:** 2013-03-01

**Authors:** Valentina Donà, Marcello Ventura, Michela Sali, Alessandro Cascioferro, Roberta Provvedi, Giorgio Palù, Giovanni Delogu, Riccardo Manganelli

**Affiliations:** 1 Department of Molecular Medicine, University of Padua, Padua, Italy; 2 Institute of Microbiology, Università Cattolica del Sacro Cuore, Rome, Italy; 3 Department of Biology, University of Padua, Padua, Italy; Fundació Institut d’Investigació en Ciències de la Salut Germans Trias i Pujol. Universitat Autònoma de Barcelona. CIBERES, Spain

## Abstract

PPE represent a peculiar family of mycobacterial proteins characterized by a 180 aminoacids conserved N-terminal domain. Several PPE genes are co-transcribed with a gene encoding for a protein belonging to another family of mycobacterial specific proteins named PE. Only one PE-PPE couple has been extensively characterized so far (PE25-PPE41) and it was shown that these two proteins form a heterodimer and that this interaction is essential for PPE41 stability and translocation through the mycobacterial cell wall. In this study we characterize the PE11-PPE17 couple. In contrast with what was found for PE25-PPE41, we show that PPE17 is not secreted but surface exposed. Moreover, we demonstrate that the presence of PE11 is not necessary for PPE17 stability or for its localization on the mycobacterial surface. Finally, we show that the PPE domain of PPE17 targets the mycobacterial cell wall and that this domain can be used as a fusion partner to expose heterologous proteins on the mycobacterial surface.

## Introduction

One of the most intriguing features of the genomes of slow growing mycobacteria is the presence of a high number of genes encoding for members of two peculiar protein families named PE and PPE. The members of these families are characterized from highly conserved N-terminal domains of about 110 or 180 aminoacids, respectively, typically containing the motif PE or PPE at the beginning of their amino acidic sequence, after which they are named [1]. Some members of these protein families are represented by a sole PE or PPE domain, while most of them present a second larger C-terminal extension which can be unique or belong to one of several subclasses [2]. Although some PE and PPE have been shown to be involved in the modulation of the immune response and/or to be essential for virulence [3], [4], [5], [6], their precise function has been elucidated or proposed only for a few of them [7], [8], [9].

All the PE and PPE proteins characterized to date were shown to be surface exposed or secreted [10], [11]. Interestingly, almost all PE nor PPE do not present canonical secretion signals and recently it has been demonstrated that both the N- and the C-terminus of the PE domain contain the information necessary to drive the translocation of this family of proteins [12], [13], [14]. In a recent study we showed that the catalytic domain of the secreted lipase LipY is fused to a PE domain in *Mycobacterium tuberculosis*, to a PPE domain in *Mycobacterium marinum* and to a canonical signal peptide in *Mycobacterium gilvum*, suggesting that these domains are interchangeable modules used by mycobacteria to target proteins to the bacterial surface [15]. Secretion of several PE and PPE is dependent on the type VII secretion system ESX-5, although the molecular mechanism of the transport process is still unknown [10], [16].We also reported that the PE domain of one of the better characterized PE proteins, PE_PGRS*33*, was able to drive the surface localization of MPT64 when it was fused at its C-terminus [12], [13]. The expression of this chimeric protein on the surface of a recombinant *Mycobacterium bovis* BCG increased its protection against *M. tuberculosis* infection, opening the possibility to exploit the function of these proteins to develop an improved vaccine against tuberculosis [17].

**Figure 1:**
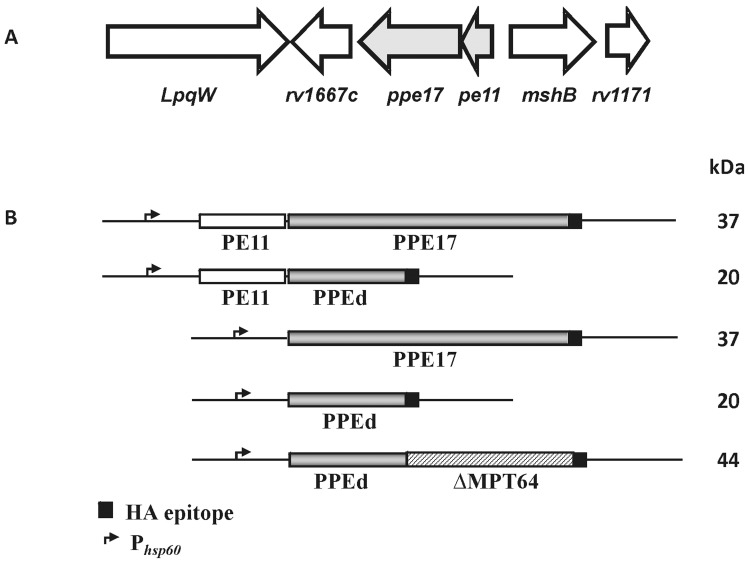
Maps of the PE11-PPE17 encoding region and of their derivatives used for this study. A) Map of the *M. tuberculosis* genomic region encoding PE11 and PPE17; B) Maps of the plasmids used to express PE11, PPE17 and MPT64 derivatives. The gene encoding PE11 is shown in white, the gene encoding PPE17 is shown in grey, the gene encoding the leaderless MPT64 is shown in stripes, while the 9 amino acid HA epitope is shown in black. The molecular weight of the PPE-derived proteins encoded by the relative genes is shown.

An interesting feature of the PPE-encoding genes is that they are often preceded by a gene encoding for a PE and, at least in case of PE25-PPE41, the PE and the PPE domains of these two proteins were shown to interact forming a heterodimer essential for the stability and/or the secretion of the PPE [14], [18], [19]. In this study, we investigate the properties of another PE-PPE couple: PE11-PPE17 (Fig. 1A). We chose these two proteins since their structural genes are strongly induced following surface stress [20] and PPE17 was recently shown to interact with Toll-like receptor-2 resulting in downstream activation of nuclear factor-κβ and HIV-1 LTR trans-activation [21]. Here we demonstrate that PPE17 is stable and surface exposed, even when expressed in the absence of the cognate PE. We also demonstrate that the PPE domain of PPE17 contains the information necessary for secretion and anchorage to the cell wall and that it can be used as a fusion partner to express antigens on the mycobacterial surface.

## Materials and Methods

### Ethics Statement

All procedures involving the use of animal were approved by the Ethical Committee of the Catholic University of the Sacred Heart, Rome.

### Bacterial Strains, Media and Growth Conditions

*M. smegmatis* mc^2^155 [22] and *M. bovis* BCG (Pasteur)were grown at 37°C in Middlebrook 7H9 (liquid medium) or 7H10 (solid medium; Difco), supplemented with 0.05% v/v Tween 80 (Sigma-Aldrich) and 0.2% v/v glycerol (Sigma-Aldrich). *M. bovis* BCG cultures were also added with ADN (2% glucose, 5% BSA, 0,85% NaCl). Strains processed for proteinase K degradation assay, and cell subfractioning were grown in Sauton (Difco). For cloning procedures *E. coli* strain HB101 was grown in Luria Bertani medium (LB) [23]. Hygromycin (Roche) was used at a final concentration of 100 µg ml^−1^ (solid media) or 50 µg ml^−1^ (liquid media) for *M. smegmatis* and at a final concentration of 200 µg ml^−1^ for *E. coli*.

### DNA Manipulation

All genes expressed in this work were amplified from the *M. tuberculosis* H37Rv chromosomal DNA with Pfu DNA polymerase (Stratagene). For the amplification of the sequence encoding HA-tagged PPE17 or its domain PPE domain (PPEd) with and without co-expression of PE11, an upper primer was designed containing an *Xba*I restriction site immediately before the start codon of the structural gene of PPE17 or PE11, respectively (RP93, RP91) (Table S1). The lower primer for the amplification of the whole gene encoding PPE17 was designed to remove the stop codon of the gene which was fused to the HA coding sequence and a stop codon followed by an *Xba*I restriction site(RP561) (Table S1). The same strategy was used to design the lower primer for the amplification of the sequence encoding the PPEd (RP560) (Table S1). All fragments were cloned into pMV10-25 [12], in order to place the transcription of the HA-tagged proteins under the control of the strong mycobacterial promoter P*_hsp60_* obtaining the following replicative plasmids: pVD28 (PE11-PPE17-HA), pVD27 (PE11-PPEd-HA), pVD31 (PPE17-HA), and pAL27 (PPEd-HA) (Table S2). The correct orientation of the inserted fragments was verified by PCR.

In order to develop a surface expression system based on the PPEd, we constructed the mycobacterial expression vector pAL26, where the sequence encoding this domain was placed under the control of P*_hsp60_* and upstream of an in frame polylinker to facilitate cloning. At this purpose, the 567 bp sequence encoding the PPEd plus the polylinker was amplified using the primers RP233 and RP234. The upper primer was designed to contain an *Xba*I site immediately before the start codon of the PPE coding sequence, while the lower primer was designed to have a polylinker containing *Bam*HI, *Pac*I and *Nco*I restriction sites before a stop codon and a *Kpn*I site. This fragment was transcriptionally fused to the P*_hsp60_* present in the shuttle vector pMV10-25 [24] digested by *Nhe*I and *Kpn*I (Tables S1 and S2).

To construct the translational fusion between PPEd and the mycobacterial antigen Mpt64, a fragment encoding Mpt64, deprived of its first 23 aminoacids (aa) (Δ-MPT64) tagged with the HA epitope was excised from pSTE2 [12], [13] and cloned into pAL26. Briefly, vectors pSTE2 and pAL26 were digested by *Bam*HI and *Nco*I and the respective fragments were separated by agarose gels, purified and ligated to obtain pAL29 (Tables S1 and S2).

### Electroporation

Electroporation of mycobacteria was performed as previously described [25]. Briefly, mid-exponential cultures were extensively washed in 10% glycerol and concentrated approximately 40-fold. One hundred µl of concentrated cells were mixed with 1 µg of DNA and transferred to 0.2 cm gap cuvettes (Eppendorf). Samples were electroporated using an Electroporator Gene Pulser Transfection Apparatus (Biorad; capacitance 25 µF; voltage12.5 kV cm^−1^; resistance 200 Ω). After the pulse, the cells were diluted in 900 ml of liquid medium, incubated for 3 h (*M. smegmatis*), or 24 h (*M. bovis* BCG) and then plated on selective solid medium.

### Protein Samples Preparation

Protein samples were prepared as previously described [13]. Briefly, mid-exponential cultures were separated from culture supernatants by centrifugation, and secreted proteins were precipitated from culture supernatants with 10% TCA (w/v). Cells were washed with PBS and thereafter subjected to proteinase K degradation, Genapol extraction or subcellular fractionation as described below. Proteins samples were boiled and separated by SDS-PAGE as described below.

### Proteinase K Degradation Assay

Proteinase K degradation assay was performed as previously described [12]. Briefly, bacteria were grown for 14 h starting from an OD_600_ of 0.1 in 20 ml of medium. Cells were washed once in TBS buffer (Tris HCl pH 7.5, NaCl 150 mM, KCl 3 mM) and resuspended in 1 ml of the same buffer. Each sample was divided in two identical aliquots. One aliquot was treated with proteinase K (Sigma-Aldrich) up to a concentration of100 µg ml^−1^, whereas the other was left untreated and incubated for 30 min at 4°C. The reaction was stopped by the addition of 1X complete EDTA-free protease inhibitor (Roche). Subsequently, samples were washed once in TBS and resuspended in TBS plus loading buffer 5X (sucrose 50% w/v, SDS 10% w/v, 0.3 M Tris HCl pH 6.8, bromophenol blue 0.05%w/v, β-mercaptoethanol 5% v/v) or subjected to subcellular fractionation as described below. Finally, samples were boiled for10 min to allow bacterial lysis and loaded on a polyacrylamide gel in equal amounts. Treated and untreated samples were processed in parallel using the same procedure to allow their comparison. Each experiment was performed at least twice with different biological samples.

### Subcellular Fractionation

Subcellular fractionation was performed as previously described [12], [13] with some modifications after proteinase K treatment, bacterial pellets were resuspended in PBS 1X/phenyl methane sulphonyl fluoride (Sigma-Aldrich, PMSF) and subjected to sonication to lyse the cells. The lysates were centrifuged at 1000×g at 4°C to precipitate cellular debris and unlysed cells. Supernatants were transferred to fresh tubes and sedimented at 27.000×g for 30 min at 4°C to recover cell wall-associated proteins. Once again, the supernatant was precipitated at 100.000×g for 2 h to separate cytoplasmic membrane from cytosolic fraction. Cytosolic proteins were subsequently concentrated on Amicon centrifugal filters (cutoff 3 kDa) to a final volume of 1 ml. Pellets were washed once after each step of centrifugation in PBS/PMSF 1 mM and finally resuspended in 1 ml of PBS plus Loading buffer 5X. Finally, samples were boiled for 5 min before being separated on polyacrylamide gels and subjected to Western blotting as described below.

### SDS-PAGE and Immunoblot

SDS-PAGE was performed according to standard protocols. Briefly, proteins were separated on 10%, polyacrylamide gels [23], and subsequently transferred to polyvinylidene fluoride membranes (PVDF; Bio-Rad) by Western blotting. Proteins were visualized by immunoblotting using monoclonal antibodies directed against the HA epitope (Anti-HA.11; Covance, dilution 1∶2000), or GFP (Chemicon; dilution 1∶2500). Secondary goat anti-mouse (Santa Cruz Biotechnology; dilution 1∶2000) horseradish peroxidase conjugates were used to detect proteins. The West Dura Signal Kit (Pierce) was used to develop the chemiluminescent signal. Image acquisitions and quantifications were performed using a Versadoc Imaging System (Bio-Rad) and Quantity One4.2.3 software (Bio-Rad).

### Enzyme-linked Immunosorbent Assay with Whole Cells of *M. smegmatis* or *M. bovis* BCG

The assay was performed as previously described [26] with some modifications: cells were grown to an OD_600_ of about 0.8, harvested by centrifugation at 3000×g for 10 minutes at room temperature, washed twice in TBST buffer (50 mM Tris-HCl pH 8.0, 150 mM NaCl, 1 mM MgCl_2_ and 0.05% Tween 80). Pellets were finally resuspended in 1 ml of TBST and divided into two identical aliquots. One aliquot was treated with proteinase K (Sigma-Aldrich) 100 µg ml^−1^, whereas the other was left untreated and incubated for 30 min at 4°C. The reaction was stopped adding 1X complete EDTA-free protease inhibitor (Roche). Samples were then centrifuged at 4°C for 5 minutes at 3000×g and washed with 500 µl of TBST. Finally pellets were resuspended in 100 µl of NaHCO_3_ 50 mM, pH 9,6 to yield a cell concentration of about 1×10^9^ cells ml^−1^. One hundred µl of the so obtained cell suspensions were transferred to each well of a 96-wellsmicrotitre plate (NUNC-Immuno Maxi Sorp Surface, Nalge Nunc International). After 24 h of incubation at 4°C, the microplate was centrifuged and the supernatant discarded. Samples were then blocked with 200 µl of 3% powdered skim milk in TBST for 90 minutes at 37°C (*M. smegmatis*) or at room temperature (*M. bovis* BCG), and subsequently washed once with 200 µl of TBST. The primary antibody (a monoclonal anti -HA.11, Covance, or a polyclonal anti-MPT64 mouse anti-serum) was diluted in 1% powdered skim milk in TBST (1∶10000 dilution for anti-HA and 1∶6400 for anti-MPT64) and 100 µl added to each well. After an incubation of 1 h at 37°C (*M. smegmatis*) or at room temperature (*M. bovis* BCG), the wells were washed three times with 200 µl of TBST. The secondary antibody (alkaline phosphatase conjugate goat anti-mouse, Santa Cruz Biotechnology) was diluted 1∶5000 in TBST containing 1% powdered skim milk and 100 µl were added to each well. Samples were incubated for 1 h at 37°C (*M. smegmatis*) or at room temperature (*M. bovis* BCG). After 4 washing steps with 200 µl of TBST, and one with TBS, 200 µl of a solution of *p*-nitrophenyl phosphate (Sigma), diluted in Tris-HCl pH 8.0 to a final concentration of 1 mg/ml, was added to each well and incubated until the development of a pale yellow colour. The reaction was stopped adding 50 µl of 3 M NaOH to each well. Absorbance at 405 nm was measured with a microplate reader (Sunrise, Tecan).

### Evaluation of the Protective Activity of Recombinant BCG

Groups of C57Bl/6 mice were immunized subcutaneously with 5×10^6^ CFU of BCG PPE-ΔMpt64, BCG PPE or, as a control, BCG Pasteur on day 0. Ten-weeks following the immunization, vaccinated and control mice were infected aerosolizing about 100 CFUs of *M. tuberculosis* Erdman using a Middlebrook chamber (Glas-Col) as described previously [27]. The vaccinated and control mice were sacrificed 28 days after challenge and bacterial colonization of lung and spleen tissues assessed as described earlier [28]. Briefly, to assess the bacterial growth *in vivo*, five mice per group were sacrificed, and the lungs and spleens were removed aseptically and homogenized separately in 5 ml of 0.04% Tween 80-PBS using a Seward Stomacher 80 blender (Tekmar). The homogenates were diluted serially in the Tween-PBS solution, and 50-µl aliquots were plated on Middlebrook 7H11 agar (Difco) containing 2-thiophenecarboxylic acid hydrazide (2 µg/ml). The number of CFUs in the infected organs was determined after 14 to 21 days of incubation at 37°C in sealed plastic bags.

## Results

### Construction of Mycobacterial Strains Expressing HA-labelled PPE17

The gene encoding PPE17, as several other PPE genes, is co-transcribed with a gene encoding a PE domain (PE11). To determine PPE17 localization, and to assess whether co-expression with PE11 plays a role in PPE17 stability or localization, we constructed a series of replicative mycobacterial expression vectors, in which the gene encoding PPE17 or just its N-terminal PPE domain (PPEd) fused to the HA epitope coding sequence (to facilitate detection), were expressed either in the presence or in the absence of the gene encoding PE11 (Fig. 1B). Each construct was placed under the transcriptional control of the strong mycobacterial promoter P*_hsp60_*. The resulting plasmids were then introduced by electroporation in *M. bovis* BCG and *M. smegmatis* mc^2^155. Protein extracts of the resulting strains were analysed by Western blot to assess the expression of the recombinant proteins. We found that PPE17 was not secreted into the culture supernatant of *M.* bovis BCG (Fig. S1) or extracted by the non-ionic detergent Genapol in *M. smegmatis* (Fig. S2).

### Surface Localization of PPE17 does not Depend on the Presence of PE11

**Figure 2:**
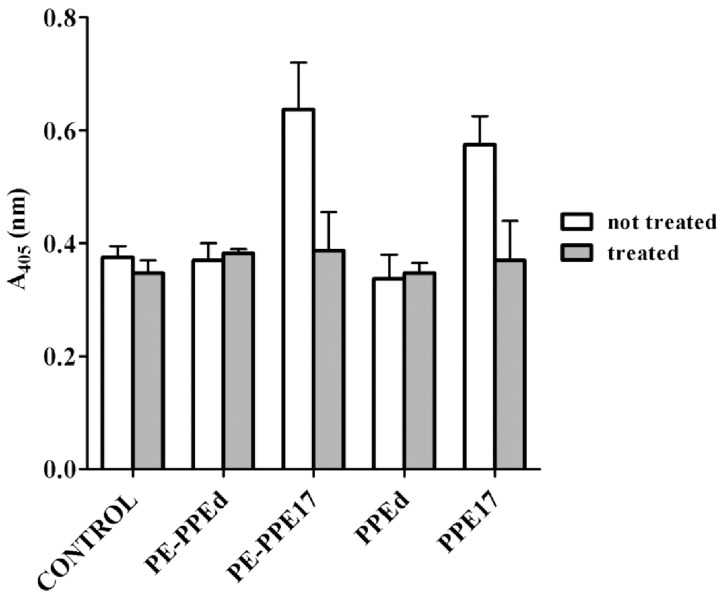
Whole-cell ELISA of *M. bovis* BCG strains expressing different proteins. Cell cultures of *M. bovis* BCG were treated (grey) or not treated (white) with proteinase K to allow degradation of surface exposed proteins. Control represents wt *M. bovis BCG*. Proteins were detected using monoclonal antibodies against HA.

The four *M. bovis* BCG strains containing the first four constructs shown in Figure 1 were grown in liquid media, and divided into two equal aliquots. One aliquot of each strain was subjected to proteinase K degradation. The resulting samples were then used to perform an ELISA assay on whole cells. The ELISA was developed as described previously [20] with an anti-HA primary antibody and a secondary antibody linked to a horseradish peroxidase. After the addition of the substrate we were expecting to detect absorbance at 405 nm for recombinant proteins exposed on the mycobacterial surface in the untreated samples, which should not be detectable in the respective samples previously treated with proteinase K. We could detect a signal above background for PPE17 regardless of PE11 co-expression (Fig. 2). Moreover, in both cases, PPE17 was sensitive to proteinase K degradation, further confirming its surface exposure. However, we could not detect any signal deriving from PPEd, suggesting that this moiety may be imbedded inside the cell wall structure, as we already reported for the PE domain of PE_PGRS*33* [12].

**Figure 3:**
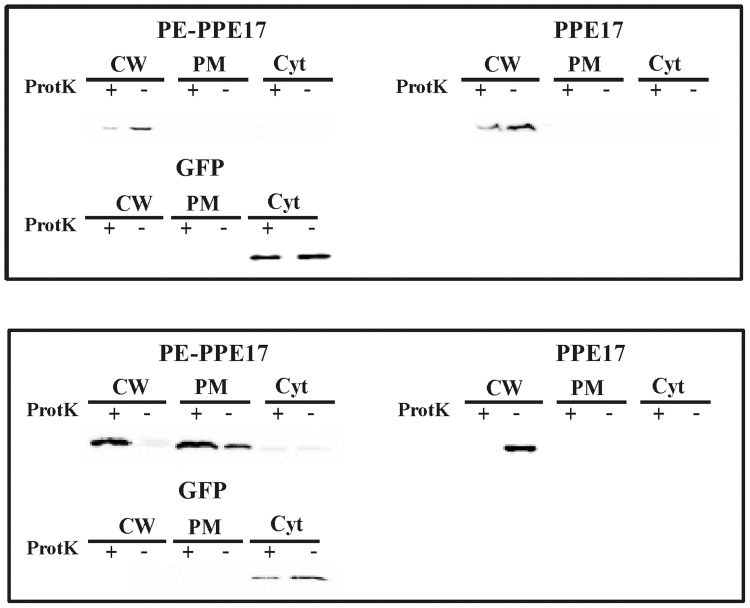
Subcellular fractionation analysis of *M. bovis* BCG and *M. smegmatis* expressing PPE17. Western blot analysis of different cellular fractions of *M. bovis* (A) or *M. smegmatis* (B) strains expressing PPE17 in presence or absence of PE11. CW: cell wall; PM plasma membrane; Cyt: cytoplasm;+treated with proteinase K; -: not treated with proteinase K. Proteins were detected using monoclonal antibodies against HA or GFP.

To further corroborate these results we repeated the proteinase K degradation assay on *M. bovis* BCG strains expressing PPE17. After the treatment all samples were lysed by sonication and subjected to subcellular fractionation. Finally, protein samples were separated on an SDS page gel and analysed by Western blot with a monoclonal antibody against the HA epitope. The intracellular protein GFP was used as a control. As shown in Figure 3A, PPE17 mainly localized in the cell wall fraction regardless of PE11 co-expression. In all samples it was sensitive to proteinase K degradation confirming the results shown in the ELISA assay. Since in *M. bovis* BCG the chromosomal copy of the gene encoding PE11 might complement the absence of this gene in the expression plasmid, we repeated this experiment in *Mycobacterium smegmatis*, which does not encodes close homologs of PE11 or PPE17: as shown in Figure 3B, also in this species PPE17 mainly localized in the cell wall fraction regardless of PE11 co-expression and was sensitive to proteinase K degradation.

### Construction and Characterization of Mycobacterial Strains Expressing Heterologous Proteins on their Surface

**Figure 4:**
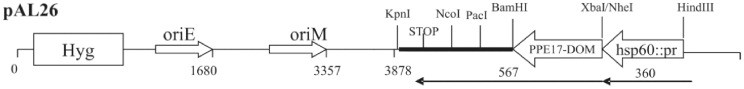
Map of pAL26. Shuttle expression vector designed to facilitate the expression of chimeric proteins fused to the PPE domain of PPE17.

In order to develop a mycobacterial expression system for surface localization of chimeric proteins based on the PPEd, we constructed an expression vector in which the sequence encoding this domain was placed downstream of P*_hsp60_* and upstream of an in-frame polylinker to facilitate cloning (pAL26) (Fig. 4).

**Figure 5:**
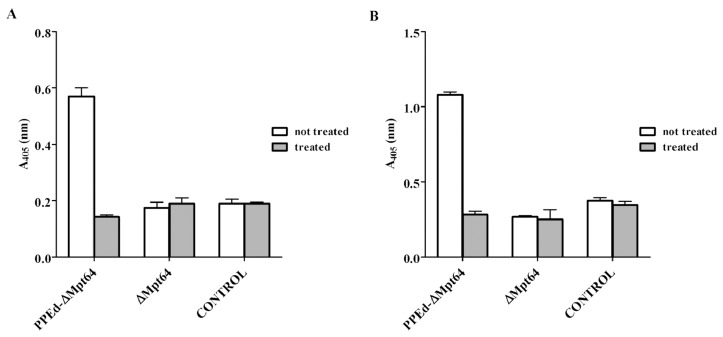
Whole-cell ELISA of *M. smegmatis* and *M. bovis* BCG expressing PPEd-ΔMPT64. Cell cultures of A) *M. smegmatis* or B) *M. bovis* BCG were treated (grey) or not treated (white) with proteinase K to allow degradation of surface exposed proteins. Control represents wt *M. smegmatis* or *M. bovis* BCG. Proteins were detected using monoclonal antibodies against HA.

**Figure 6:**
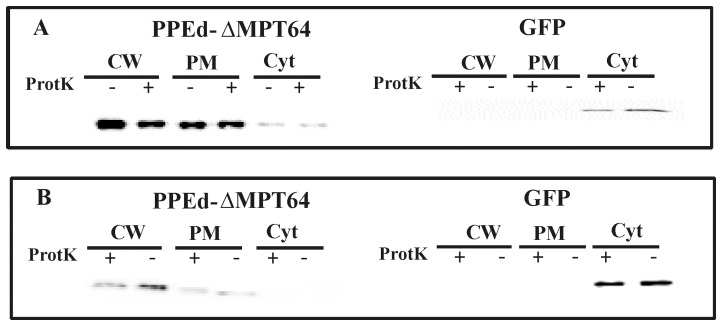
Subcellular fractionation analysis of *M. smegmatis* and *M. bovis* BCG expressing PPEd-ΔMPT64. Western blot analysis of different cellular fractions of A) *M. smegmatis* and B) *M. bovis* BCG strains expressing PPEd-ΔMPT64. CW: cell wall; PM plasma membrane; Cyt: cytoplasm;+treated with proteinase K; -: not treated with proteinase K. Proteins were detected using monoclonal antibodies against HA or GFP.

The sequence encoding MPT64 (a protective antigen absent in several *M. bovis* BCG strains) [17], [27] deprived of its signal sequence (ΔMPT64) was cloned in frame with the sequence encoding the PPE domain and the HA epitope. The resulting vector was electroporated in *M. smegmatis* and *M. bovis* BCG. The resulting strains were subjected to proteinase K degradation assay followed by whole-cell ELISA. As shown in Figure 5, the fusion protein was clearly detectable in both *M. smegmatis* and *M. bovis* BCG, whereas strains expressing ΔMPT64 showed a signal comparable to the negative control, represented by strains not expressing MPT64, demonstrating that PPEd was driving the localization of the chimeric protein on the mycobacterial surface. Moreover, in both mycobacterial species, the fusion protein was sensitive to the proteolytic activity of the proteinase K, confirming that PPEd-ΔMpt64 is surface exposed. To further corroborate these data theses trains were also subjected to proteinase K degradation assay followed by subcellular fractionation. As shown in Figure 6 in both organisms the chimeric protein was mainly found in the cell wall fraction and was sensitive to degradation.

### Evaluation of the Protective Activity Induced by rBCG Expressing PPEd-ΔMPT64 in the Mouse Model of Tuberculosis

**Figure 7:**
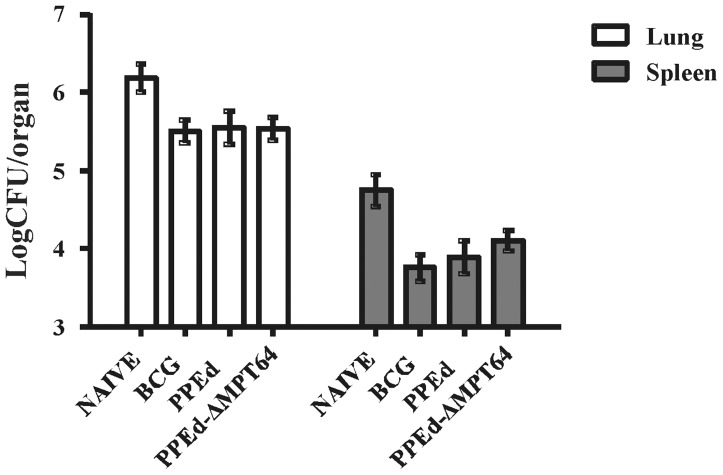
Protection after challenge with virulent *M. tuberculosis.* Protective activity induced by BCG expressing PPEd-ΔMPT64 in two independent experiments. Immunized and control mice were infected 10 weeks post-immunization with *M. tuberculosis* Erdman. Twenty-eight days later mice were sacrificed and lung and spleen bacterial load were determined by CFU counting.

**Figure 8:**
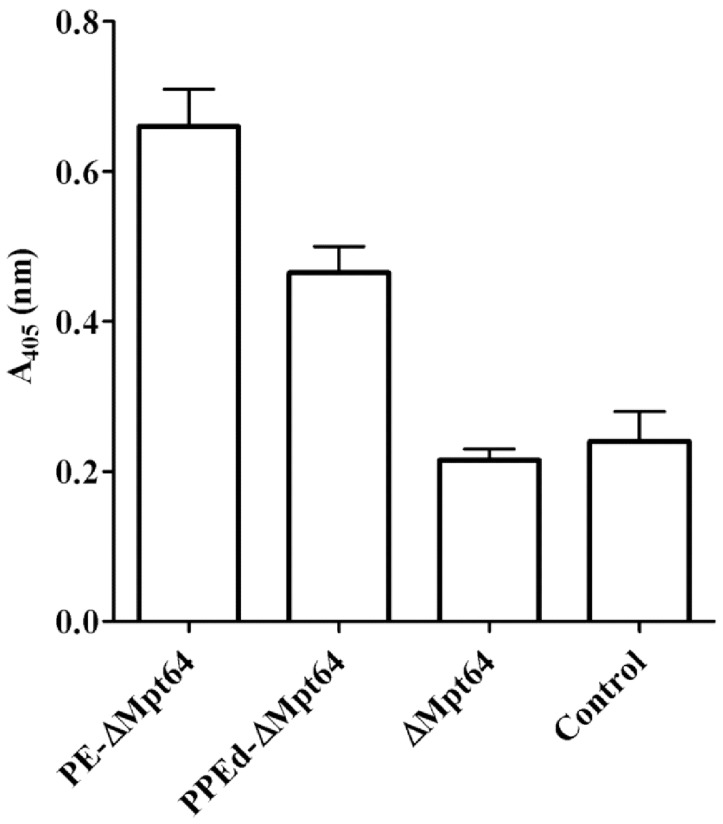
Whole-cell ELISA of *M. bovis* BCGstrains expressing PE-ΔMPT64 or PPEd-ΔMPT64. Controls are represented by *M. bovis* BCG wt or expressing intracellular ΔMpt64. Proteins were detected using a polyclonal antibody against MPT64.

In order to evaluate if the expression of surface exposed PPEd-ΔMPT64 on can improve the protection against *M. tuberculosis* infection of the vaccine *M. bovis* BCG strain, female C57Bl/6 mice were immunized s.c. with *i)* the recombinant BCG expressing the PPEd-ΔMPT64 chimeric protein; *ii)* the recombinant BCG expressing PPEd and *iii)* the parental BCG strain following standard procedures. Ten weeks after vaccination, mice were infected by aerosol with a low dose of *M. tuberculosis* Erdman. Four weeks later the mice were sacrificed and the bacterial loads in the lung and spleen tissues were assessed by CFU counting. As shown in Figure 7, all mice previously immunized with BCG showed a significant reduction in lung CFUs compared to non-immunized mice, but no differences were observed between mice immunized with recombinant or parental BCG. Similar results were observed in the spleen. Taken together these results indicate that expression of the candidate antigen MPT64 on the surface using the PPEd-based delivery system does not provide enhanced anti-TB immunity. In a previous study we showed that a recombinant *M. bovis* BCG strain expressing the surface exposed fusion protein between the PE domain of PE_PGRS*33* and ΔMPT64 does improve protection against *M. tuberculosis* infection compared to the parental strain. A potential explanation for this difference is that a higher amount of MPT64 is expressed on the surface of the *M. bovis* BCG strain expressing PE-ΔMPT64 than in that expressing PPE-ΔMPT64. In order to test this hypothesis, we performed a whole-cell ELISA on *M. bovis* BCG strains expressing the two different chimeras. As shown in Figure 8, the signal of the strain expressing PE-ΔMPT64 was higher than that obtained from the strain expressing PPEd-ΔMpt64, suggesting that the exposure of the PE-based chimera was more efficient than that of the PPE-based chimera.

## Discussion

PPE proteins are divided into 5 subfamilies (I–V) depending on their evolutionary lineage [2]. The single member of subfamily I, 4 out of 10 members of subfamily II, 3 out of 6 members of subfamily III, and 9 out of 26 members of subfamily IV are encoded by genes located immediately downstream of a gene encoding a PE protein. With two exceptions (Rv2769c: PE27, 275 aa and Rv3018a: PE27a, 28 aa) the PE genes associated with PPE genes encode for proteins of about 120 aa containing only the PE domain [2], which is likely able to interact with PPE domains. The PE and PPE domains of the well characterized PE25-PPE41 couple have been previously shown to form a heterodimer essential for the stability and/or the secretion of the cognate PPE [19]. Moreover, the interaction between several PE and PPE domains not encoded by adjacent genes has also been predicted [29].

In a previous study we determined that the PE domain of PE_PGRS*33* is responsible for its surface localization [12], [13] and that it can be used as a fusion partner to expose heterologous antigens on the *M. bovis* BCG surface leading to an increase of its protective activity against *M. tuberculosis* infection [17].

The aim of this work was to assess the role of the PPE domain of PPE17 and of its cognate PE protein (PE11) in PPE17 cellular localization. PPE17 is a 346 aminoacids protein containing a 180 aa PPE domain followed by a large domain exhibiting the GxxSVPxxW motif of unknown function which typically characterizes the members of PPE subfamily III [2]. The genes encoding PE11 and PPE17 are strongly induced in *M. tuberculosis* following surface stress and PPE17 was recently shown to interact with Toll-like receptor-2 resulting in downstream activation of nuclear factor-κβ and HIV-1 LTR trans-activation [21]. Finally, it is worth knowing that in the genome of *M. bovis* (both wt and BCG) the PPE17-encoding gene contains a mismatch resulting in the production of a truncated protein of 543 aminoacids.

We constructed four plasmids expressing full-length HA-labeled PPE17, or just its HA-labeled PPE domain, with or without PE11 co-expression, which were introduced in *M. bovis* BCG (Fig. 1). The surface exposure of PPE17 and PPEd with or without PE11 co-expression was tested in *M. bovis* BCG through whole-cell ELISA performed on cultures previously subjected to the proteinase K degradation assay (Fig. 2). We could easily detect the presence of PPE17 on the bacterial surface regardless of PE11 co-expression, strongly suggesting that PE11 is not essential for PPE17 transport across the cell wall.

The subcellular localization of these recombinant proteins in *M. bovis* BCG was further characterized by a proteinase K degradation assay followed by subcellular fractionation, which confirmed the results obtained in the ELISA assay. PPE17 was principally found in the cell wall fraction, and was degraded by proteinase K further confirming its surface localization (Fig. 3). Surprisingly, the presence of PE11 was not required for stability or for surface localization of PPE17, in contrast to PPE41 which requires the presence of PE25 [14], [18], suggesting that different PE-PPE couples might have different molecular roles in transport and assembly. Even if we cannot exclude that in *M. bovis* BCG the chromosomal copy of the gene encoding PE11 might complement the absence of this gene in the expression plasmid, this is unlikely since PPE17 was surface exposed also when expressed in *M. smegmatis* which does not encode a PE11 close homolog (Fig. 3B).

Since overexpression from a strong promoter might affect cellular localization as well, we performed preliminary experiments with strains expressing PE11 and PPE17, whose expression was placed under the control of the weak promoter P*_Rv1818c_*: subcellular fractionation experiments gave results perfectly overlapping those obtained with strains expressing PE11 and PPE17 under the control of the strong promoter P*_hsp60_*, ruling out the possibility of artifacts due to overexpression. However, the level of expression in these strains was too low to allow protein detection in whole-cell ELISA experiments (data not shown).

In conclusion, we demonstrated that PPE17 is surface exposed regardless of the presence of PE11. We further hypothesized, that its PPE domain may contain the signal sufficient for surface localization. In order to confirm this hypothesis, we constructed a plasmid expressing a chimeric protein, in which PPEd was fused to a leaderless MPT64, a protective antigen of *M. tuberculosis* absent in several *M. bovis* BCG strains. This plasmid was then introduced in both *M. smegmatis* and *M. bovis* BCG. Whole-cell ELISA and subcellular fractionation experiments performed after proteinase K treatment confirmed that the PPEd-ΔMPT64 chimera was surface expressed in both species (Fig. 5 and 6). These data confirm that the PPE domain of PPE17, as previously shown for the PPE domain of LipY in *M. marinum* [15], and for the PE domains of PE11, PE_PGRS*33* and LipY in *M. tuberculosis* [12] contains the information necessary for directing the protein to the cell envelope and suggest that not all PPE proteins (not even those coexpressed with a cognate PE protein) require a PE partner for their surface localization.

Secretion of several PE and PPE proteins in the model organism *M. marinum* require ESX-5 [10]. Moreover, the secretion of several PE and PPE proteins sharing some immunodominant epitopes with PE19 and PPE25, whose structural genes are physically associated to ESX-5, has been recently shown to require a functional ESX-5 secretion system for their translocation across the *M. tuberculosis* envelope [4]. PE11 and PPE17 do not contain any of these shared epitopes rendering a prediction of their dependence on ESX-5 impossible at this time. Additional studies, for instance the determination of the subcellular localization of these proteins in mycobacterial mutant strains lacking the ESX-5 secretion system, are required in order to assess the relevance of this secretion system for PE11-PPE17 translocation across the mycobacterial envelope.

In a previous study we showed that surface-expression of ΔMPT64 in *M. bovis* BCG driven by the PE domain of PE_PGRS*33* caused an increase of the protective potential of this recombinant vaccine strain against virulent *M. tuberculosis* infection in mice in comparison to the parental strain [17]. These results prompted us to perform a protection study using the recombinant *M. bovis* BCG strain expressing the PPEd-ΔMPT64 chimera on its surface for immunization. Surprisingly, in this case we could not detect any increase in protection after challenge with virulent *M. tuberculosis* (Fig. 7).The fact that the amount of the PE-based chimera exposed on the bacterial surface was higher than that of the PPE-based chimera (Fig. 8), might explain the reason of this difference in the protective efficacy of the two recombinant BCG strains. At present we do not know if the difference in the amount of exposed protein was due to a difference in the efficiency of the PE and PPE domains in exporting proteins or simply to difference in the stability of the two chimeras. Alternatively, the sole PE domain of PE_PGRS*33* was shown previously to be able to elicit predominantly cell-mediated immunity and subsequent protection against challenge when expressed in a DNA vaccine [25], and might thus have adjuvant properties also when used as a fusion partner, whereas the immunogenic properties of the PPE domain of PPE17 are still unknown.

Thus, although the expression of antigens on the *M. bovis* BCG surface appears to be a promising strategy to increase the protective potential of this vaccine strain, further studies are indeed required in order to draw final conclusions on the efficacy and versatility of this approach. Further understanding of the mechanisms of transport and cell wall anchorage of PE and PPE proteins, as well as their differential immunogenic properties, will be absolutely necessary to finally reveal the role of these peculiar proteins in *M. tuberculosis* physiology and virulence, and for their biotechnological exploitation.

## Supporting Information

Figure S1 **Western blot of recombinant** ***M. bovis*** **BCG surnatants.** 1–3: *M. bovis* BCG expressing the secreted protein MPT64-HA; 4–6: *M. bovis* BCG expressing PE-PPE17-HA. 1 and 4: surnatant; 2 and 5: surnatant 1∶5; 3 and 6: proteins extracted from the pellet. Molecular weight in kDa are shown on the right. Proteins were detected using monoclonal antibodies against HA.
(JPG)

Figure S2 **Genapol surnatant from** ***M. smegmatis*** **strains expressing different proteins.** PE-PPE17-HA (1), PE_PGRS33-HA (2), delta-MPT64-HA (3). Molecular weight in kDa are shown on the right. Proteins were detected by Western blot using monoclonal antibodies against HA.
(JPG)

Table S1 **Primers used in this study.**
(DOC)

Table S2 **Plasmids used in this study.**
(DOCX)
