# Supplementary figures and images for: The PPE Domain of PPE17 Is Responsible for Its Surface Localization and Can Be Used to Express Heterologous Proteins on the Mycobacterial Surface
Source: PLoS One. 2013 Mar 1;8(3):e57517. doi: 10.1371/journal.pone.0057517 (PMC3586085; doi:10.1371/journal.pone.0057517)

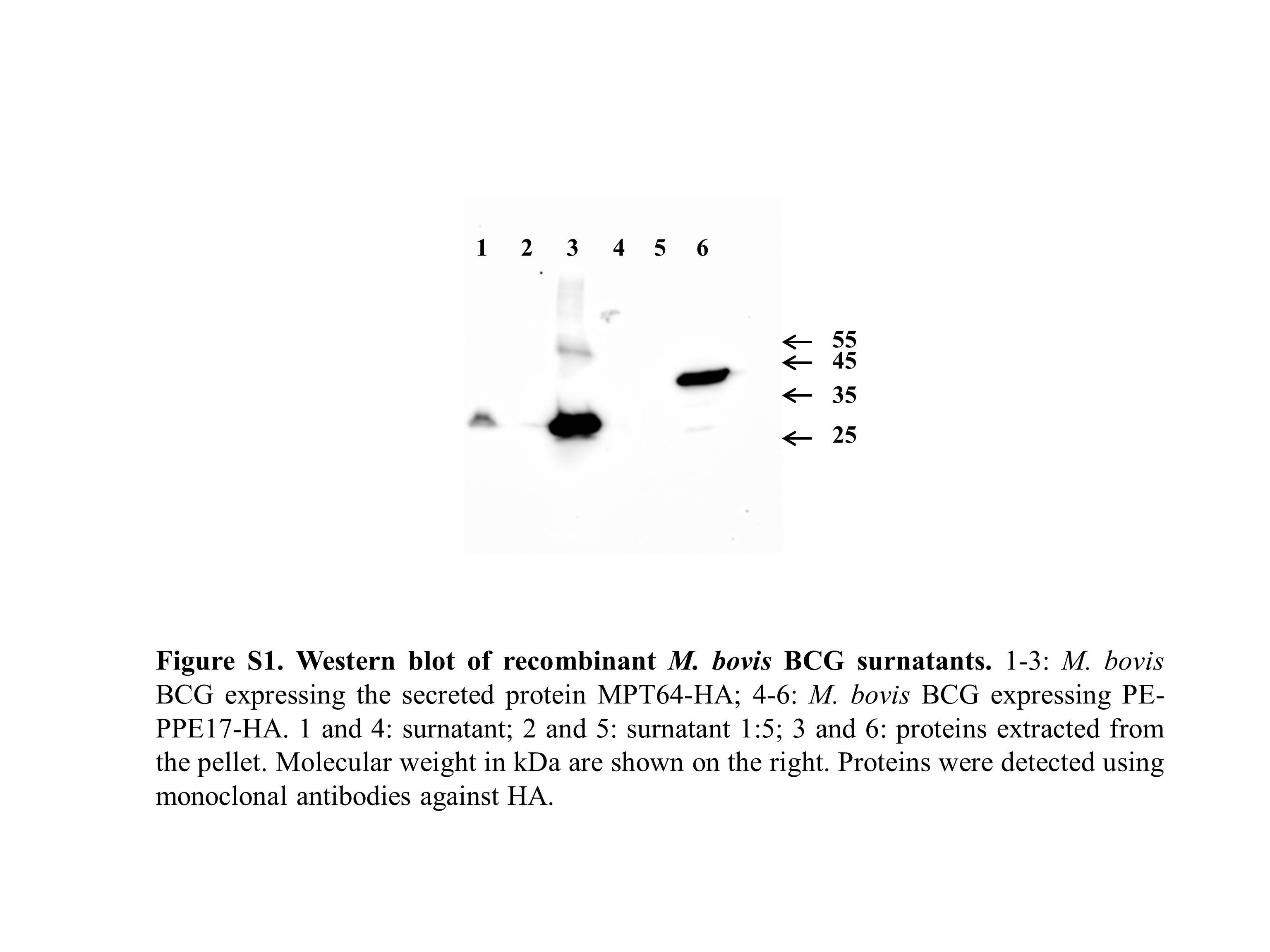

Supplement: Figure S1 — Western blot of recombinant M. bovis BCG surnatants. 1–3: M. bovis BCG expressing the secreted protein MPT64-HA; 4–6: M. bovis BCG expressing PE-PPE17-HA. 1 and 4: surnatant; 2 and 5: surnatant 1∶5; 3 and 6: proteins extracted from the pellet. Molecular weight in kDa are shown on the right. Proteins were detected using monoclonal antibodies against HA. (JPG) [file pone.0057517.s001.jpg]

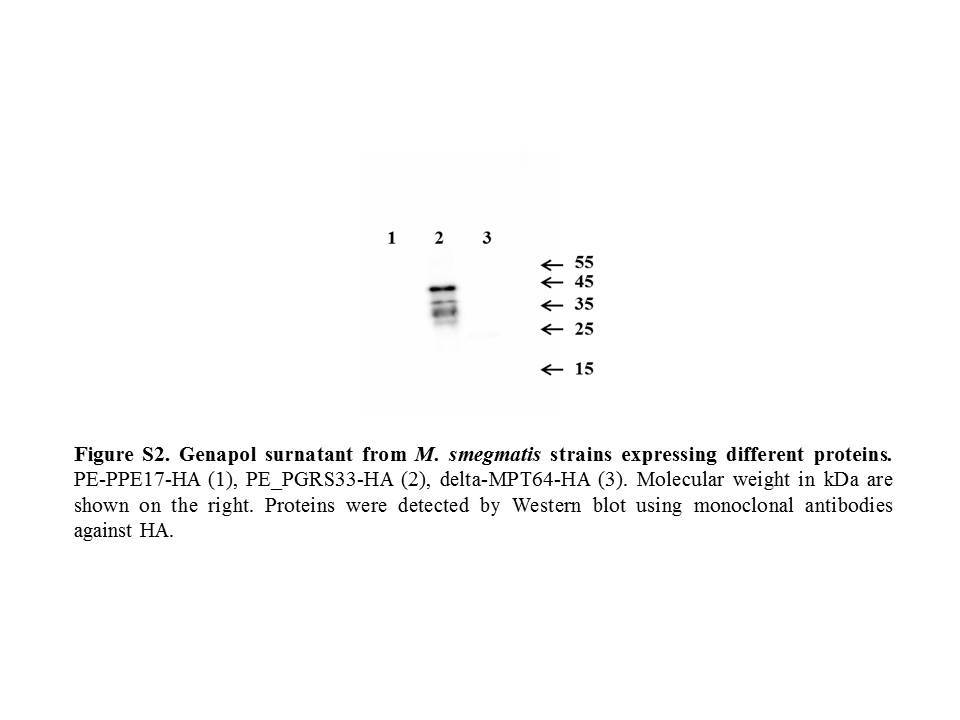

Supplement: Figure S2 — Genapol surnatant from M. smegmatis strains expressing different proteins. PE-PPE17-HA (1), PE_PGRS33-HA (2), delta-MPT64-HA (3). Molecular weight in kDa are shown on the right. Proteins were detected by Western blot using monoclonal antibodies against HA. (JPG) [file pone.0057517.s002.jpg]
