# Supplementary material for: The PPE Domain of PPE17 Is Responsible for Its Surface Localization and Can Be Used to Express Heterologous Proteins on the Mycobacterial Surface
Source: PLoS One. 2013 Mar 1;8(3):e57517. doi: 10.1371/journal.pone.0057517 (PMC3586085; doi:10.1371/journal.pone.0057517)
Supplement: Table S1 — Primers used in this study. (DOC) [file pone.0057517.s003.doc]

| **Primer** | **Sequence^a^** | **Product^b^** |
| --- | --- | --- |
| RP91 | ACTCTAGAGTGTCTTTTGTCACCACACGG | PE11-PPE17-HA; PE11-PPE17d-HA (U) |
| RP93 | ACTCTAGAATGGATTTCACAATTTTTCCGCCG | PPE17-HA; PPE17d-HA (U) |
| RP561 | TGTCTAGATCA*AGCATAATCAGGAACATCATA*CGGATAGTTAATTAATCCAGCGGCGGGTGA | PE11-PPE17-HA - PPE17-HA (L) |
| RP560 | TGTCTAGATCA*AGCATAATCAGGAACATCATA*CGGATGTTAATTAAAGCCAGCGCGCCCGG | PE11-PPE17d-HA - PPE17d-HA (L) |
| RP233 | ATTCTAGAATGGATTTCACAATTTTTCCG | PPE17d-polylinker (U) |
| RP234 | AAGGTACCTACCCATGGTTAATTAAGGATCCAGCCAGCGCGCCCGG | PPE17d-polylinker (L) |

**Table S1. Primers used in this study.**

^a^ Restriction sites are underlined, nucleotides encoding the HA epitope are in italic.

^b^ Protein encoded by the gene amplified by the corresponding primers. U: upper primer; L: lower primer
