# Supplementary material for: The PPE Domain of PPE17 Is Responsible for Its Surface Localization and Can Be Used to Express Heterologous Proteins on the Mycobacterial Surface
Source: PLoS One. 2013 Mar 1;8(3):e57517. doi: 10.1371/journal.pone.0057517 (PMC3586085; doi:10.1371/journal.pone.0057517)
Supplement: Table S2 — Plasmids used in this study. (DOCX) [file pone.0057517.s004.docx]

**Table S2.** **Plasmids used in this study.**

| **Plasmid** | **Expressed protein(s)** | **Primers used for construction** |
| --- | --- | --- |
| pVD26 | PPE17d-HA | RP93/RP560 |
| pVD27 | PE11-PPE17d-HA | RP91/RP560 |
| pVD28 | PE11-PPE17-HA | RP91/RP561 |
| pVD31 | PPE17-HA | RP93/RP561 |
| pAL26 | PPE17d-polylinker | RP233/RP234 |
| pAL29 | PPE17d-Δ−Mpt64-HA | NA |
